# Erosive Benign Fibro-osseous Lesion of the External Auditory Canal

**DOI:** 10.7759/cureus.2135

**Published:** 2018-02-01

**Authors:** Ganesh Vihapure, Vivek Dokania, Nimish Thakral

**Affiliations:** 1 ENT Department, Krishna Institute of Medical sciences, Karad; 2 SHRI B M Patil Medical College, SHRI B M PATIL MEDICAL COLLEGE VIJAYAPUR

**Keywords:** benign fibro-osseous lesion, external auditory canal

## Abstract

The term fibro-osseous lesion encompasses a spectrum of disorders ranging from inflammatory to neoplastic that microscopically exhibit a connective tissue matrix containing formless trabeculae of compact bone. Characteristically, they are located over healthy bone, from which they are abruptly differentiated. The majority of the lesions arise from the maxillofacial region; the occurrence of a lesion in the external auditory canal (EAC) being extremely rare as is in our case. The lesions present with a range of symptoms ranging from conductive hearing loss, Eustachian tube obstruction to bone erosion that develop due to the mass effect. We report a case of a 35-year-old male patient who presented with insidious onset left aural fullness, decreased hearing followed by intermittent mucopurulent discharge from the ear, who was eventually diagnosed with a benign fibro-osseous lesion of the external auditory canal.

## Introduction

An erosive benign fibro-osseous lesion of the external auditory canal (EAC) is rarely seen. We found only four cases during our review of literature spanning the last 15 years [1]. The term fibro-osseous lesion is a generic term encompassing a wide range of disorders ranging from inflammatory to neoplastic that microscopically exhibit a connective tissue matrix, which contains a variable amount of mineralized substance that may be osteoid or cementum-like. These lesions present with a wide array of symptoms that usually result from the mass effect exerted by the lesion.

## Case presentation

A 35-year-old Indian male presented with insidious onset left ear fullness and decreased hearing since six months followed by intermittent mucopurulent discharge from the left ear. The discharge later turned mucopurulent with foul odour. No associated vertigo, imbalance, tinnitus, facial asymmetry, altered sensorium, nausea, or vomiting was reported. There was no history of aural trauma, ear procedure, or surgery.

Otoscopy showed an opalescent lesion at the bony-cartilaginous junction of the left external auditory canal. The lesion was completely occluding the left EAC and obscuring the view of the tympanic membrane. On gentle probing, the lesion was firm, non tender, and non-bleeding. Tuning fork test showed a negative Rinne test on the left side and Weber lateralized towards the left ear. A pure tone audiogram showed a flat curve with moderate conductive hearing loss (50-55 dB) in frequencies ranging from 250 Hz through 8000 Hz on the left side.

**Figure 1:**
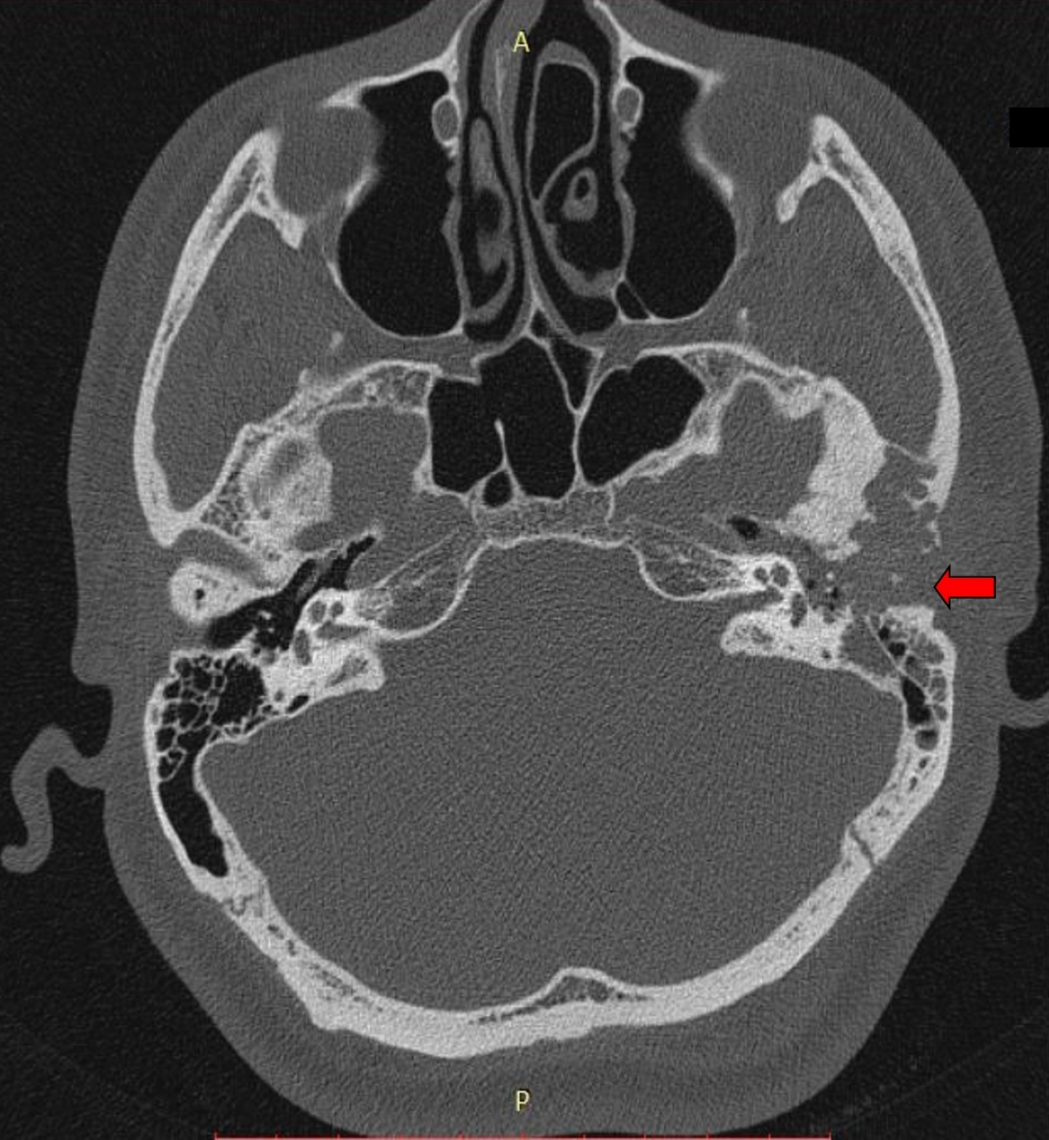
High-resolution computed tomography (HRCT) of temporal bone in axial view showing lesion occupying left external auditory canal and middle ear cleft, and eroding adjacent areas.

High-resolution computed tomography (HRCT) of the temporal bone revealed ill-defined, mildly hyperintense soft tissue opacification of the left middle ear cavity with bulging into the cartilaginous portion of the left EAC. Bony septae within the roof of the left temporomandibular joint were absorbed, associated with severe cortical erosion of the anterior bony wall of the left EAC. The lesion was noted abutting the incus and the malleus. Erosion of mastoid air cells septae and opacification of the mastoid antrum were noted. The sinodural plate appeared intact (Figure 1).

**Figure 2:**
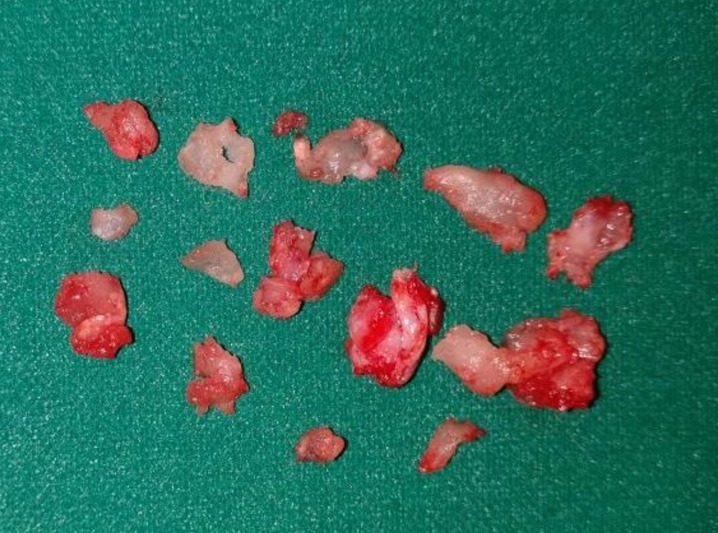
Excised surgical specimen in pieces.

Canal wall down modified radical mastoidectomy with meatoplasty was undertaken under general anaesthesia. The lesion along with granulation tissue was removed from the external auditory canal, mastoid cavity, and middle ear in a piecemeal fashion (Figure 2) and was sent for histopathological examination. Necrotic incus remnant was removed and ossiculoplasty was undertaken. Gel foam was placed in situ, post-auricular closure suturing was done in three layers, and mastoid dressing was applied. The dressing was removed on the third postoperative day, and the external suture was removed on the tenth postoperative day, displaying a healthy wound. No recurrence or complication was noted until the seven-month postoperative follow-up.

**Figure 3:**
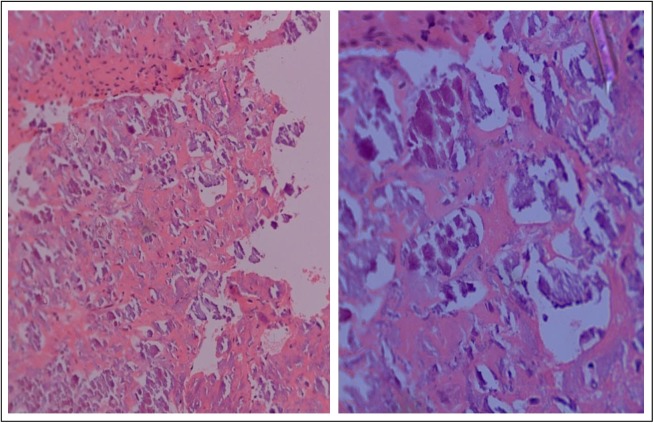
Hematoxylin and eosin stained low (100X) and high resolution (400X) histological images showing bony trabeculae with extensive calcification enmeshed in a fibrocollagenous stroma.

Histopathological findings revealed bony trabeculae with extensive calcification enmeshed in a fibrocollagenous stroma (Figure 3). These findings were consistent with a diagnosis of benign fibro-osseous lesion.

## Discussion

Benign fibro-osseous lesions of the external auditory canal are often confused with osteomas and exostosis, and hence we must know about each of the above conditions in detail to arrive at a proper diagnosis. Osteomas of the external auditory canal usually arise from the tympanosquamous or tympanomastoid suture as solitary bony growths that are pedunculated [2]. Exostoses are seen as a response to local irritant phenomena. They are multiple, usually bilateral, and symmetrical with a broad implantation base in the deepest regions of the external auditory canal [2].

Benign fibro-osseous lesions have an unknown etiology. However, Macarenco et al. postulate that they result from chronic inflammation secondary to local trauma or chronic minor local injury [3]. They are characteristically located over healthy bone from which they can be readily differentiated.

Histopathologically, an encapsulated fibrous tissue or connective tissue with interwoven bony trabeculae is seen frequently; there is also a presence of small blood vessels. Cartilaginous tissue is not seen and hence local ossifying phenomenon can be ruled out [4]. Calcification of various degrees is also noted [5]. There is a varying degree of mineralization in the form of woven bone or cementum-like basophilic structures [6]. As the histologic picture is nonspecific, it helps in ruling out malignancy but is of limited value in guiding treatment [7]. The biologic activity of the lesion is primarily of importance in guiding the treatment of the patient. A computed tomography (CT) scan provides valuable insight regarding the biological activity of the tumor, as in our case, the tumor or expansile lesion resulting in localized erosive phenomenon [7].

Clinically, the patients present with progressive ear pain and hearing loss, which can later result in ear discharge if the mass gets infected. The ear pain is of variable intensity. Hearing loss is most often conductive in nature and is dependent upon the size of the mass, involvement of the middle ear ossicles, and obstruction of the external auditory canal.

There is no gold standard treatment or specific protocol in the treatment of benign fibro-osseous lesion. As these lesions are slow growing in the majority of cases, observation is sufficient for asymptomatic cases. In symptomatic cases, multiple biopsies may be required for a definitive diagnosis. Surgical exploration and curettage are required in these cases to prevent further complications such as meningitis or encephalitis that can result from localized erosion [6].

## Conclusions

Benign fibro-osseous lesion of the external auditory canal is extremely rare and its erosive variant remains under-recognized worldwide. HRCT of the temporal bone provides valuable insight regarding the biological activity of the tumor and the extent of lesion for preoperative planning. A histological picture aids in ruling out malignancy and reaching a most probable diagnosis.
